# Genome Sequence of a Hepatitis E Virus of Genotype 3e from a Chronically Infected Kidney Transplant Recipient

**DOI:** 10.1128/genomeA.01156-13

**Published:** 2014-01-30

**Authors:** Valérie Moal, Audrey Ferretti, Patricia Devichi, Philippe Colson

**Affiliations:** a Aix-Marseille Université, URMITE UM 63 CNRS 7278 IRD 198 INSERM U1905, Facultés de Médecine et de Pharmacie, Marseille, France; b Assistance Publique-Hôpitaux de Marseille, Hôpital Conception, Centre de Néphrologie et Transplantation rénale, Marseille, France; c IHU Méditerranée Infection, Pôle des Maladies Infectieuses et Tropicales Clinique et Biologique, Fédération de Bactériologie-Hygiène-Virologie, Centre Hospitalo-Universitaire Timone, Assistance Publique-Hôpitaux de Marseille, Marseille, France; d Assistance Publique-Hôpitaux de Marseille, Hôpital Conception, Centre d’Investigation Clinique, Marseille, France

## Abstract

Hepatitis E virus (HEV) is an emerging cause of acute and chronic hepatitis in immunocompromised patients in Europe. We report the genome sequence of a genotype 3e HEV from a chronically infected kidney transplant recipient in southeastern France, the second HEV genome sequence from a transplant recipient and the first of subtype 3e.

## GENOME ANNOUNCEMENT

Hepatitis E virus (HEV) was first described in 1983 in Russia following investigation of a hepatitis outbreak in a Soviet military camp in Afghanistan (1), and the first genome sequence was described in 1991 (2, 3). Four genotypes and 24 subtypes were defined (4). Genotypes 1 and 2 are anthroponotic only and circulate in developing countries, whereas genotypes 3 and 4 circulate in humans and mammals, mostly swine (5, 6). HEV-3 is the majority genotype in Western developed countries, where hepatitis E was considered until recently as imported from areas of hyperendemicity, but it turned out that most cases are autochthonous (5, 6). Moreover, the existence of a porcine reservoir and foodborne transmission were demonstrated for genotypes 3 and 4 HEV (6–8). Southern France has been identified as an area of hyperendemicity (5). HEV RNA was detected there in pig liver sausages, which can transmit HEV when eaten uncooked (5, 6, 9–11). In our geographical area, southeastern France, HEV infection is of particular concern in kidney transplant recipients, among whom the estimated incidence was 1.2% or higher and the progression rate to chronic infection was 80% (12).

In 2009, HEV RNA of genotype 3e was obtained from a 46-year-old French kidney transplant recipient followed up for his graft in our institution. This patient received a first kidney transplant in 2004 and presented clinically with a silent acute HEV infection in 2009. Diagnosis relied on a moderate increase of liver enzyme levels and then the detection of anti-HEV IgM/IgG and HEV RNA in serum (12). The patient ate cooked wild boar meat during the 9 prior weeks. He recovered 17.5 months later, after reduction in his immunosuppressive drug dosage.

Viral RNA was extracted from the patient serum using the EZ1 Virus Minikit v2.0 on the BioRobot EZ1 Workstation (Qiagen, Courtaboeuf, France). A near full-length genome sequence (7,132 nucleotides) was obtained by reverse transcription-PCR followed by Sanger population sequencing using 12 sets of primers covering the HEV genome. Overlapping sequences were assembled manually and by the CLC bio software (CLC bio) by mapping on closely related genomes. Phylogeny reconstructions based on the whole-genome sequence or a fragment of open reading frame (ORF) 2 demonstrated that the HEV genotype was 3e. This genotype was described in pigs and humans in Japan and Europe (including France, Spain, the United Kingdom, and Greece) (4) and was involved in a minority of infections in France, including infections in solid organ transplant recipients (12–14). In our center, HEV-3e was mostly detected in other kidney transplant recipients (12, 15). The HEV genome described here is the first of subtype 3e, and the second overall, recovered from solid organ transplant recipients. The highest nucleotide similarity (89%) was found in sequences recovered in Germany and Japan from pigs (GenBank accession numbers FJ998015 and AB248520, respectively), while the closest HEV genome recovered from a human was from a Japanese patient (AB291958). Coding regions corresponding to ORFs 1, 2, and 3 were found to encode peptides of 1,696, 617, and 122 amino acids, respectively. Continuing HEV surveillance in human and animal reservoirs is critical to gain a better knowledge of the diversity and epidemiology of this virus.

### Nucleotide sequence accession number.

The sequence is available in GenBank under accession no. KF922359.
